# Melkersson–Rosenthal syndrome misdiagnosed as recurrent Bell’s palsy: a case report and review of literature

**DOI:** 10.1186/s13223-020-00508-z

**Published:** 2021-01-09

**Authors:** Yared Zenebe Zewde

**Affiliations:** Department of Neurology, School of Medicine, College of Health Sciences, Addis Ababa University, P.O. Box 41690, Addis Ababa, Ethiopia

**Keywords:** Melkersson–Rosenthal syndrome, Orofacial edema, Facial palsy, Fissured tongue, Bell’s palsy, Ethiopian

## Abstract

**Background:**

Melkersson–Rosenthal syndrome (MRS) is a rare neuro-mucocutaneous disorder of unknown cause, clinically characterized by a triad of recurrent facial palsy, orofacial swelling, and fissured tongue. It is frequently seen in females in their second and third decades of life. MRS is diagnosed based on clinical features and it is rarely possible to observe all the classical triad symptoms at the same time. The disorder may cause recurring peripheral facial palsy that is wrongly diagnosed as recurrent Bell’s palsy

**Case presentation:**

A 25-year-old female patient was presented to the neurology clinic of Tikur Anbessa Specialized Hospital in Addis Ababa complaining of recurrent left-side peripheral facial weakness, facial swelling and fissured tongue of 5 days duration. Her past medical history was positive for similar symptoms, for which she was diagnosed with Bell’s palsy and received oral corticosteroid treatment. On examination left side lower facial swelling with flat naso-labial fold and fissured tongue were identified. After excluding other mimickers, she was diagnosed with Melkersson–Rosenthal syndrome and completely recovered with high dose of corticosteroid treatment.

**Conclusion:**

Melkersson–Rosenthal syndrome may present with the classic triads of symptoms, but mostly it shows an incomplete clinical pattern. Therefore, when clinicians including allergists encountered patients with facial swelling and facial palsy, they should have to consider MRS in their differential diagnosis and specifically assess for recurrent facial palsy and fissured tongue. Unlike true angioedema, the facial swelling in MRS often develops gradually and it might cause permanent swelling with cosmetic disfigurement from multiple relapses, which can be prevented by early detection and timely initiation of treatment.

## Background

Melkersson–Rosenthal Syndrome (MRS) is a rare, chronic non-caseating granulomatous neuro-mucocutaneous disorder that is often mis- and under-diagnosed [1]. Although the exact cause and pathogenesis of MRS has not been identified yet, genetic predisposition, allergic reactions, hypersensitivity, autoimmune and microbial reactions have been hypothesized [2]. Its classic presentation with a triad of recurrent facial nerve paralysis, facial and/or lip swelling, and fissured tongue (lingua plicata) are found in less than a quarter of patients [3]. MRS presentation is mimicked by a wide spectrum of conditions including orofacial granulomatous inflammation associated with sarcoidosis and Crohn’s disease. Other causes of recurrent facial weakness such as hypothyroidism, herpes zoster oticus, Lyme disease, and Bell’s palsy should be also considered in the differential diagnosis of MRS [1, 4].

We report a 25-year-old right-handed, female patient who was referred to our neurology outpatient clinic in Tikur Anbessa Specialized Hospital with a complaint of difficulty to close her left eyelid with facial swelling on the same side for the previous 5 days. In the past she had similar symptoms that was diagnosed as a recurrent Bell’s palsy and symptoms resolved after corticosteroid treatment. On examination, fissured tongue was identified in addition to facial asymmetry and facial edema. After excluding other causes, she was diagnosed with MRS and completely recovered with a high dose of corticosteroid treatment. Early detection and treatment might prevent cosmetic complications from recurrent swelling and nerve injury.

## Case presentation

A 25-year-old black female patient was referred from a nearby town and presented to the neurology clinic of Tikur Anbessa Specialized Hospital in Addis Ababa. Her main complaint was an insidious onset painless swelling of her left upper lip, which progressed to involve her left cheek. She had difficulty to close her left eyelid for the previous 5 days. Her past medical history was positive for two episodes of same side peripheral facial weakness. During the first attack in 2016, her symptoms resolved spontaneously without any treatment. But when it recurred 2 years later, she visited a nearby clinic and was diagnosed as a case of recurrent Bell’s palsy, which showed a complete resolution following corticosteroid treatment. She did not recall any cause or inciting factor that had induced her symptoms. She had no history of recent infection of the ear, nose, throat, sinus or dental structures. She denied any recent history of trauma to the head or neck region, skin rash or sensory symptoms. She had no personal history of sarcoidosis, Crohn’s disease, autoimmune conditions or any other chronic illness, and her family history was negative for similar conditions. She denied any history of food allergy or any other forms of allergic reactions in the past.

**Fig. 1:**
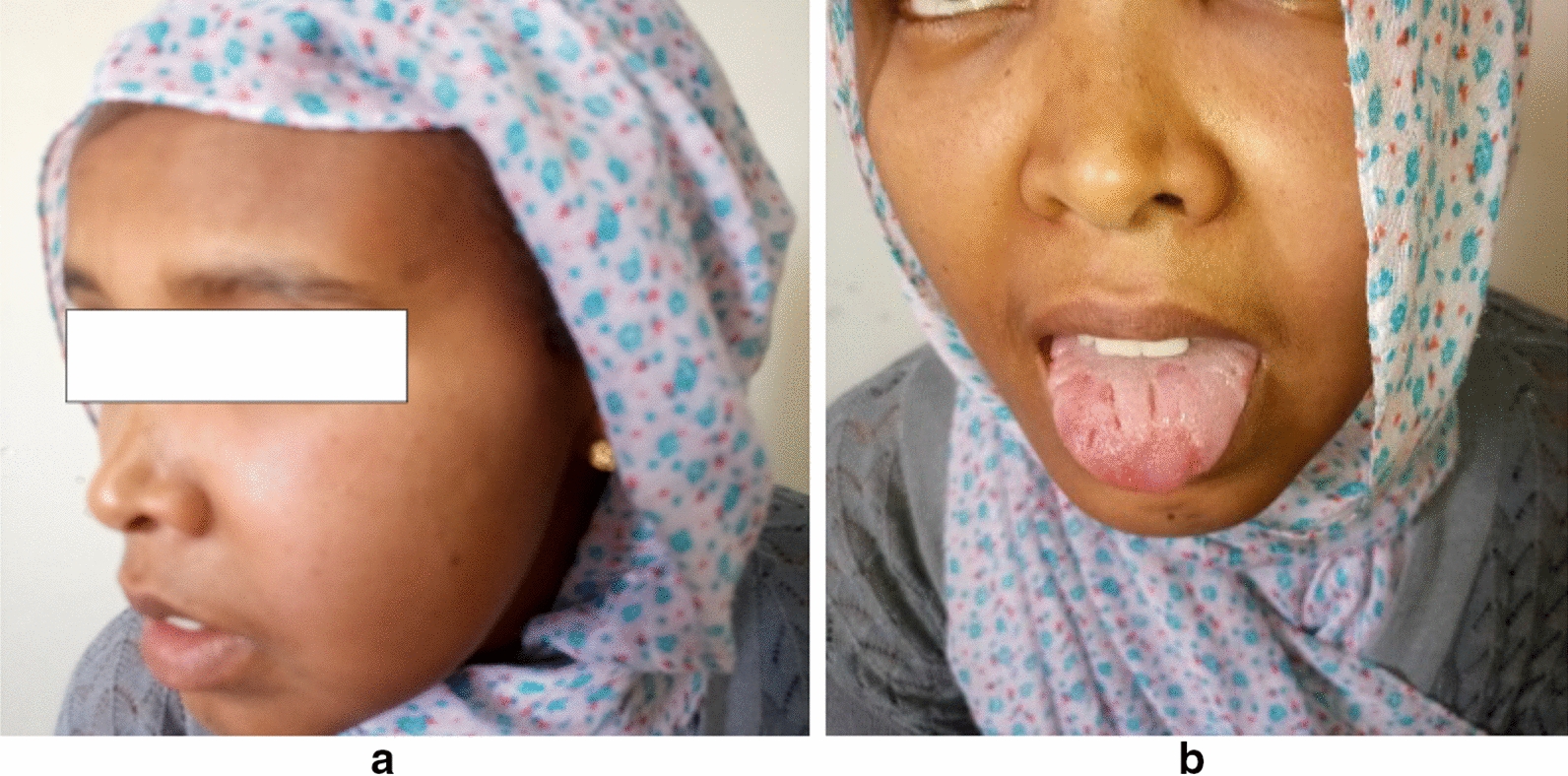
Photo of a patient with **a)** diffuse swelling of the left cheek and lower eyelid, **b)** fissured tongue and mild flattening of left naso-labial fold

**Fig. 2:**
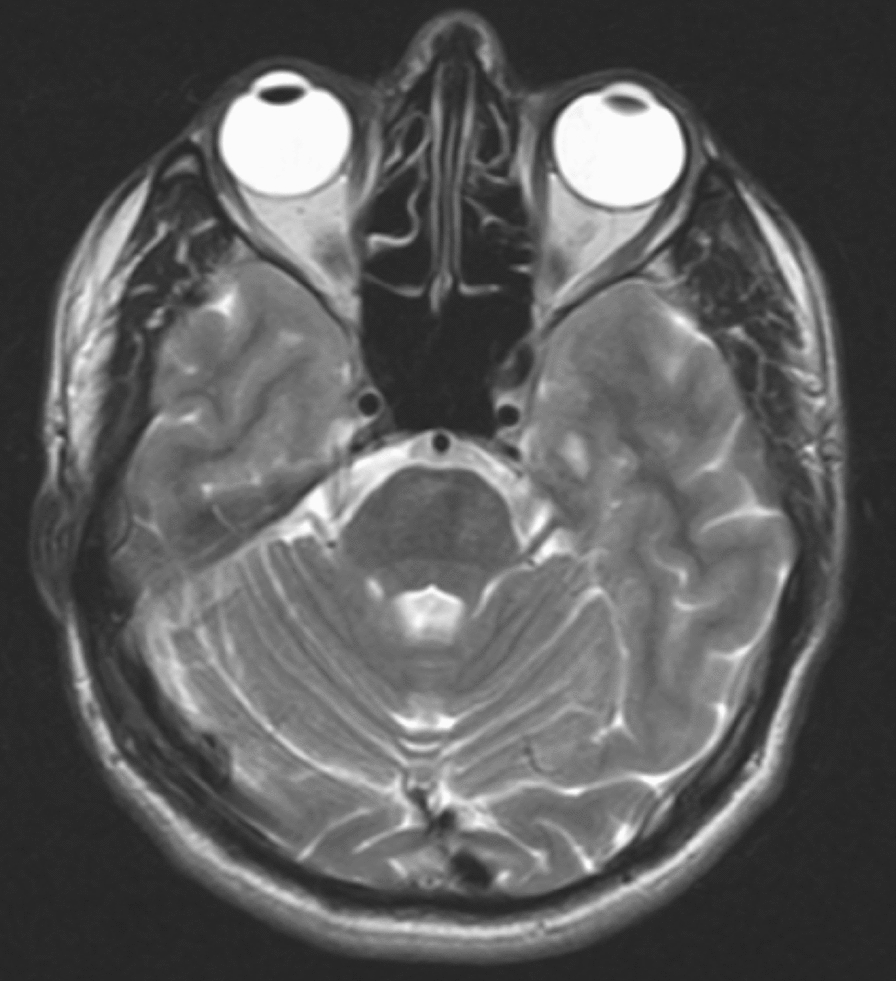
Axial T2-weighted brain MRI showed a clear course of left facial nerve

On examination, she has a diffuse, non-pitting left cheek swelling with peri-orbital fullness (Fig. 1a), mild left naso-labial fold flattening and fissured tongue (lingua plicata) (Fig. 1b). Her vitals were normal and no sensory change in the face detected. The rest of her examination was non-remarkable. Clinical and laboratory evaluations for a possible cause of recurrent peripheral facial palsy including diabetes mellitus, syphilis, Guillain–Barre syndrome, hypothyroidism, leukemia or other tumors was non-revealing. Magnetic resonance imaging (MRI) of the head showed a normal brain parenchyma and clear course of the seventh nerve (Fig. 2).

With a high index of clinical suspicion for MRS, she was started on oral Prednisone 1 mg/kg/day with gradual tapering over 2 weeks. On the third day of treatment, her facial edema started to subside and by the end of second week all symptoms resolve and she was followed in the outpatient clinic.

## Discussion and conclusions

Melkersson–Rosenthal Syndrome is a neuro-mucocutaneous syndrome belonging to the orofacial granulomatosis which was first reported by Melkersson in 1928, where a woman with intermittent peripheral facial palsy and lip edema was documented [5]. Later in 1931, Rosenthal completed the triad of the syndrome by adding the presence of fissured tongue [6].

The incidence of MRS in the general population is not clearly defined but it was estimated to be around 0.08%. This rare disease is more frequently diagnosed in the second and third decades of life and females are relatively more affected [7]. Similarly, this study reported a young female in her early 20’s at the time of symptom onset.

The etiology of MRS remains uncertain but it was postulated that genetics, infectious, and immunologic factors may play an etiologic role [1]. There are reports of MRS associated with viral and other bacterial Infections, autoimmunity, neurotrophic factors, atopy and hypersensitivity reactions to various antigens including food additives like monosodium glutamate which may have pathogenesis effect but not confirmed as etiologic agent [3].

The classic clinical presentation with a triad of recurrent peripheral facial paralysis, recurrent or persistent orofacial edema, and fissured tongue is observed in only 8% to 25% of MRS patients [3]. Recurring lip and/or orofacial edema is the most common presenting symptom by 80% to 100% of patients [4]. The facial edema is generally painless, unilateral and most often occurs in the upper lip. Less frequently, edema may involve cheeks, palate, gingiva, tongue, pharynx, larynx, and periorbital region [8]. Facial paralysis is observed in 47%–90% of cases and can be transient or sometimes permanent. It is most often unilateral but rarely can be bilateral and recurrence rate is around 10% [1]. Oligosymptomatic forms are reported in around 50% of cases, while the combination of orofacial edema and facial palsy was seen by Chan et al. in 22% of cases [9]. In 40% of MRS patients, fissured tongue (lingua plicata) is identified [10]. The classic triads are observed in less than quarter of MRS patients, so this make clinical diagnosis very difficult and led to the wrong diagnosis like in our patient, where she was diagnosed with recurrent Bell’s palsy in the past.

However, the presence of a similar but milder event in the past, indicating that the underlying disease process has been there for some time and that it appears to be episodic and recurrent in nature. The diagnosis of MRS is difficult as there are no acknowledged diagnostic criteria or biomarkers to test. But many authors agreed that MRS is a clinical syndrome that is diagnosed by constellations of signs and symptoms with no need for further investigation. But in oligo-symptomatic cases, the diagnosis is confirmed by histopathological examination, showing non-caseating granulomas [11]. However, as shown in our case, searching for subtle clinical features like a fissured tongue might help us to complete the clinical picture when symptoms are incomplete.

Currently there is no standard therapy for MRS and the treatment is mainly symptomatic. Management options to control facial swelling and facial paralysis includes antihistamines and corticosteroid administrations, either systemic or intralesional, might be the initial treatment choice [12]. Reconstructive surgery might be available for MRS patients with refractory lip edema [13]. Prophylactic decompression of the facial nerve through its bony canal should be considered for patients with frequent facial palsy to prevent progression and future attacks [14].

In conclusion, this case report highlights the importance of considering MRS in the differential diagnosis when patients with recurrent peripheral facial palsy and/or angioedema are encountered. Clinicians should have to ask for recurrent facial swelling and weakness and examine for tongue abnormalities, as early diagnosis and treatment might prevent permanent disabilities from cosmetic disfigurement.

## Data Availability

All are available in the manuscript.

## Abbreviations

MRS: Melkersson–Rosenthal syndrome
MRI: Magnetic resonance imaging
